# Development of Physical Fitness among the Top 10 Boys and Girls in Sport Schools: A 10-Year Cohort Analysis

**DOI:** 10.3390/sports7100222

**Published:** 2019-10-12

**Authors:** Andreas Roth, Steffen C. E. Schmidt, Sina Hartmann, Ilka Seidel, Swantje Scharenberg, Klaus Bös

**Affiliations:** 1 Research Centre for School Sports and the Physical Education of Children and Young Adults, Karlsruhe Institute of Technology, 76131 Karlsruhe, Germany; sina.hartmann@kit.edu (S.H.); seidel@osp-niedersachsen.de (I.S.); swantje.scharenberg@kit.edu (S.S.); 2 Institute of Sports and Sports Science, Karlsruhe Institute of Technology, 76131 Karlsruhe, Germany; steffen.schmidt@kit.edu (S.C.E.S.); klaus.boes@kit.edu (K.B.)

**Keywords:** talent identification, motor test, German Motor Test

## Abstract

In this study, we aimed to measure the development of physical fitness (PF) of 10 different cohorts in grade 4 and 8 different cohorts in grade 7 at 18 sport schools of North Rhine-Westphalia, Germany. A total of 11,451 subjects (3979 female, 7472 male) aged 8–12 from the past 10 years were assessed using the German Motor Test (DMT) in grade 4. We tested 2614 subjects (1032 girls, 1582 boys) aged 11–15 from the past eight years using the DMT in grade 7. PF talents were defined as the top 10 boys and top 10 girls of each cohort. Linear regression was calculated to assess the development of PF. The PF of all subjects remained stable in grade 4 and declined in grade 7. The PF of the top 10 boys and top 10 girls increased in both grades. The improvements were stronger in grade 7 (female: rates of change (β) = 0.80; male: β = 0.76) than in grade 4 (female: β = 0.36; male: β = 0.32). Sit-ups and push-ups showed the highest change rates. The increase in PF of the top 10 boys and girls can be interpreted as a success for sport schools. Due to the increasing number of test participants, the likelihood of finding top talent increased. However, the increase in PF in the top talents was only partly explained by an increase in the number of tested individuals.

## 1. Introduction

Due to the increasing pressure to recruit talent as early as possible [1], finding efficient methods to identify potential talents among increasingly large populations is important. However, talent is a complex and dynamic matter and depends on many factors [2]. Hohmann and Seidel [3] proposed four talent criteria: juvenile performance, speed of performance development, use of performance conditions, and load tolerance. The first two criteria are based on the measurement of physical fitness (PF). The use of performance conditions is linked to the assumption that young athletes should achieve their performance with economical use of resources. In the long term, full resources should be used to achieve maximum performance. Load tolerance is understood as the potential to manage physical and psychological stress [3]. This paper addresses juvenile performance.

In North Rhine-Westphalia (NRW) in Germany, a total of 18 sport schools were established to support the sports career of potential sport talent. Candidates have to pass two selection stages to join the sport classes starting from grade 8. In grade 4, which corresponds usually to the ages of 9 to 11 years, the German Motor Test (DMT 6–18) [4] is performed to measure general PF. In grade 7, which corresponds to the age of 12 to 14 years, pupils are re-tested to measure PF development [3]. Many talent-specific test batteries are available, but these talent batteries are usually sport-specific and are not suitable for testing across several sport disciplines [5]. The DMT is a widely spread test battery that is designed to use the available materials in school settings in Germany. 

The change in PF between different time intervals has been widely discussed internationally and in Germany. A comprehensive review by Tomkinson and Olds [6] compared studies from 27 countries between 1958 and 2003 with a total of more than 25 million 6- to 19-year-old subjects in the dimension of cardiorespiratory fitness. They found that cardiorespiratory fitness first increased and then decreased, and that cardiorespiratory fitness showed an overall decrease at an annual average rate of −0.36%. In contrast, speed and strength improved slightly [7]. In a further study, the cardiorespiratory fitness of nearly one million children and adolescents from different countries was analyzed between 1981 and 2014; the results showed a moderate decline but that this decline diminished with each decade and stabilized near zero around the year 2000 [8].

Bös [9] wrote a review of many German studies from 1965 to 2002 assessing cardiorespiratory fitness, flexibility, strength endurance, fast force, and sprinting speed of several hundred thousand children aged 6 to 17 years. After a slight increase, an average decline of 10% occurred from 1975 to 2002 across all parameters. The most obvious changes were differences in flexibility and cardiorespiratory fitness. Following this review, 51 studies from 2002 to 2006 were analyzed in a second review [10] that confirmed the decline in PF. Also, Bös et al. [10] showed that the decline in children aged 6 to 11 years (5.5%) is lower than that of 12- to 17-year-olds (12.5%).

A representative German study [11] analyzed changes in the PF of children and adolescents aged 4–17 years. A baseline cohort (2003–2006; 2205 girls and 2323 boys) was compared to cohort 1 (2009–2012; 1378 girls and 1442 boys) in terms of seven test tasks widely used in Germany (stand-and-reach, standing long jump, push-ups, bicycle ergometer test, jumping sideways, backward balancing, and pins stuck). Results showed that 24 out of 52 mean values increased, but the other 28 values did not change significantly. After a decline in PF at the end of the 20th century compared to previous decades, PF, in recent years, appears to be stable or even rising in Germany.

Regarding the development of PF at sport schools, special conditions must be mentioned. First, the PF of sport pupils is above the German average. Second, the number of sport schools and the number of belonging pupils have increased over time; therefore, changes in the general PF of pupils at sport schools, as well as that of top talent, are likely. 

We aimed to analyze the development of physical fitness (PF) of 10 different cohorts in grade 4 and 8 different cohorts in grade 7 at 18 sport schools to reveal potential changes in the overall PF of sports school candidates and pupils. The top 10 PF talents in each cohort were defined and analyzed for differences in overall PF and the performance in different test tasks to track the 10-year development of talents in sport schools. 

## 2. Materials and Methods

### 2.1. Study Sample and Design

The data were obtained annually from pupils in grade 4 and 7 across 18 sports schools in NRW: Düsseldorf, Minden, Solingen, Dortmund, Münster, Cologne, Paderborn, Mönchengladbach, Dormagen, Essen, Leverkusen, Gelsenkirchen, Bochum, Bonn, Winterberg, Duisburg, Mülheim, and Bielefeld/Herford. Each sport school differed slightly in terms of talent promotion, number of applicants, catchment areas, and main sport disciplines (soccer, track and field, handball, badminton, basketball, fencing, gymnastics, hockey, judo, rowing, swimming, table tennis, tennis, volleyball, and wrestling) [12]. The tests were organized and administered by trained instructors of the sport schools and the Research Centre of School Sports and the Physical Education of Children and Young Adults (FoSS). 

The number of test participants increased during the course of the study due to the increase in the number of participating sport schools and pupils. In grade 4, measurements were recorded from November to April in 2007/2008 to 2016/2017. A total of 11,451 children (3979 girls, 7472 boys) aged 8–12 years were tested.

In grade 7, measurements were recorded from February 2009 to May 2010 to February 2016 to May 2017 leading to data from a total of 2614 children (1032 girls, 1582 boys) aged 11–15 years. The study was approved by the review board of the Institute of Sports and Sport Science, Karlsruhe Institute of Technology.

### 2.2. Measures

#### 2.2.1. Physical Fitness

DMT 6–18 is based on the differentiation of motor abilities (endurance, strength, speed, coordination, flexibility) [13] and is used to measure the PF of boys and girls in grades 4 and 7. The 1-week test-retest reliability (Pearson correlation coefficient *r*) of test tasks performed by a comparable team of trained instructors was on average 0.82 (20 m sprint: *r* = 0.90; backward balancing: *r* = 0.52; sideways jump: *r* = 0.89; stand-and-reach-test: *r* = 0.94; push-ups: *r* = 69; sit-ups: *r* = 0.78; standing long jump: *r* = 0.89; 6 min run: *r* = 0.92). The test battery was successfully checked for validity [4].

For six of the eight tasks (standing long jump, sideways jump, backward balancing, stand-and-reach test, push-ups, and sit-ups), representative data for Germany are available (norm sample). For the other two tasks (20 m sprint and 6 min run), comparative data were obtained from different samples [4].

The sprinting speed was captured via a 20 m sprint using a Brower light timing system (Brower Timing Systems, Utah 84020, Draper, UT, USA) or a stop watch. Cardiorespiratory fitness was measured with a 6 min run. The test participants had to run for six minutes around the volleyball field marked with pylons. The total distance was recorded. The strength endurance of the torso muscles was examined by the number of sit-ups achieved in 40 s on a gym mat. The Strength endurance of the upper extremities was measured by the number of push-ups achieved in 40 s on a gym mat. Speed strength of the lower extremities was examined by the distance achieved with a standing long jump. The distance from the starting line to the heel of the foot closest back after landing was measured (cm) with a fixed tape measure. Cross motor coordination under time constraint was measured by a sideways jump. The aim is to jump sideways with both legs over the center line of a field (50 × 100 cm) marked with masking tape. Backward balancing allows the assessment of gross motor coordination during dynamic precision tasks. The test participants must walk backward and keep their balance on beams 300 cm long and of width 3, 4.5, and 6 cm. The total number of steps was used for evaluation. The stand-and-reach test was used to assess the flexibility of the trunk and sciatic crural muscle group. The test person stands on a long bench and slowly bends the upper body forward. The hands are led parallel along a centimeter- scale as far down as possible and the distance reached was captured [4].

#### 2.2.2. Body Mass Index (BMI)

Height was measured barefoot to the nearest 0.1 cm using a fixed tape measure. Weight was measured standardized to the nearest 0.1 kg using a Korona Alva digital metric scale (Sundern, North- Rhine Westphalia, Germany).

#### 2.2.3. Talent Diagnostics 

PF talents were defined as the top 10 boys and top 10 girls in each cohort based on the overall *Z*-score. The formula is: *Z* = 100 + 10 × (individual value—mean of the norm sample)/standard deviation of the norm sample. No consensus exists in the discussion about how many people are considered to be talents. The increase or decrease in a selection limit directly impacts possible talent promotion [14]. The selection limit of 10 for the sport schools of NRW has a practical implication regarding high-performance sports in Germany and cannot be generalized for other countries.

#### 2.2.4. Statistical Analysis

Statistical analysis was performed using IBM SPSS Statistics version 25 (IBM, Armonk, New York, NY, USA) and the significance level was set to *p* < 0.05. Test performance was converted to *Z*-scores. The development of PF was analyzed using linear regression and rates of change (β) were calculated.

Since an increase in performance among top PF talent is likely a function of the number of tested individuals (*N*), we added post hoc analysis of covariance (ANCOVAs) to analyze which differences between the cohorts (cohort effects) can be explained by differences in *N*. 

## 3. Results

### 3.1. Descriptive Results

On average, participants from grade 4 were 9.42 years old, 1.41 m tall, and weighed 34.5 kg with an average BMI of 17.2. The values changed only slightly during the study. Top 10 boys and girls were 9.08 years old, 1.39 m tall, and weighed 31.7 kg with an average BMI of 16.2. There was no significant age effect between the cohorts among those PF talents.

In grade 7, girls (50.5 ± 9.7 kg) were slightly heavier than boys (49.3 ± 10.3 kg) (*F* = 7.4; *p* < 0.01) and had a higher BMI (19.4 ± 3.1) than boys (18.9 ± 2.9) (*F* = 17.4; *p* <0.01). The top 10 PF talents from grade 7 were 13.0 years old, 1.61 m tall, and weighed 47.3 kg with an average BMI of 18.3. No significant age effect was found between the cohorts among those PF talents.

### 3.2. Development of Physical Fitness

Overall *Z*-scores of the total sample and top 10 PF talents from grades 4 and 7 and regression coefficients are presented in Table 1.

The PF of the top 10 boys in grade 4 increased (β = 0.32; *p* < 0.01) during the study. The PF across all boys (β = −0.01; *p* = 0.825) remained stable. The PF of girls in grade 4 increased for the top 10 (β = 0.36; *p* < 0.01), but not overall (β = 0.00; *p* = 0.961). The top 10 boys in grade 7 showed a significant increase in PF (β = 0.76; *p* < 0.01) and a decrease across all participants (β = −0.29; *p* < 0.01).

**Figure 1:**
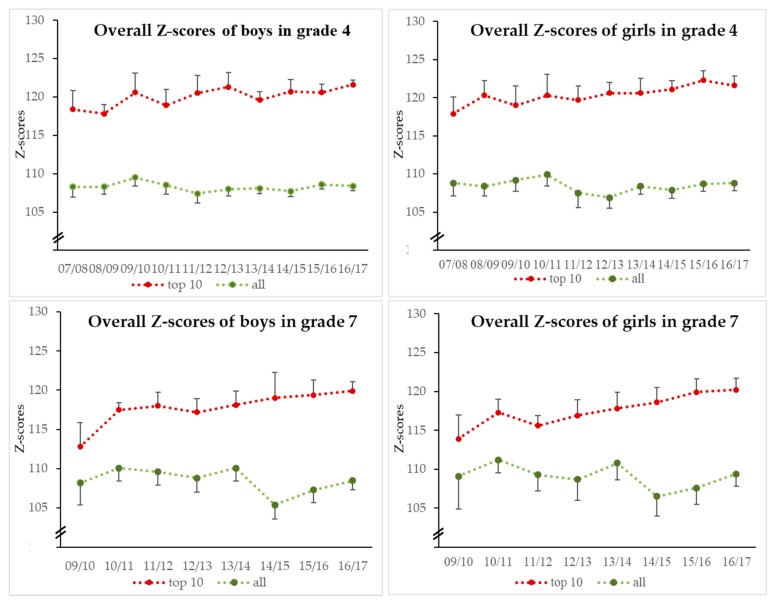
Means and 95% confidence of PF for different cohorts of boys and girls from grades 4 and 7.

The PF of the top 10 girls increased (β = 0.80; *p* < 0.01) but the average girl’s PF decreased (β = −0.24; *p* < 0.05). Differences between all test participants and the top PF talent were more pronounced in grade 4 than in grade 7. Looking at the PF talents, girls showed slightly higher change rates than boys. The development of PF among the total sample and PF talents is shown in Figure 1.

### 3.3. Development of Physical Fitness of Top 10 Boys and Girls

The *Z* scores for different tasks for PF talents from grade 4 are shown in Table A1. The β for all tasks was positive except for sprint (boys: β = −0.54; *p* < 0.01; girls: β = −0.24; *p* = 0.240). Among the boys, the highest β was observed for push-ups (β = 0.77; *p* < 0.01). For girls, the highest β was observed for sit-ups (β = 0.64; *p* < 0.01) and stand-and-reach (β = 0.64; *p* < 0.05). This corresponds to a total improvement of more than four centimeters in the stand-and-reach task and three to four repeats in the sit-up task throughout the study.

For grade 7, Z-scores of PF talents are shown in Table A2. In grade 7, the β values for overall *Z* scores were positive for boys (β = 0.76; *p* < 0.01) and girls (β = 0.80; *p* < 0.01). The rates of change were positive across all tasks. Boys achieved the highest rate of change in push-ups (β = 1.58; *p* < 0.01), whereas girls achieved the highest rate of change in sit-ups (β = 1.93; *p* < 0.01). This corresponds to a total improvement of more than seven repeats in the sit-up task and a total improvement of four repeats in the push-up task.

The *Z* scores of different tasks for all boys and girls are provided in Table A3 (grade 4) and Table A4 (grade 7). 

## 4. Discussion

The first goal of this study was to analyze the development of PF of the average pupil and the top 10 PF talents to reveal potential changes in overall PF of sports school candidates and pupils. Our study shows an increase in the PF of the top 10 talents in grades 4 and 7 in both sexes. The rates of change were higher in grade 7 than in grade 4. Looking at the average grade 4 pupil, no change occurred in PF over the course of the study; whereas for grade 7, average PF decreased for both sexes. These results are not in line with representative data from Germany [11], which show a slight increase in PF from 2003 to 2012. The reason for a declining PF among the average pupils at sport schools in NRW is possibly due to the fact that more less-talented children applied.

In the second part of the study, the development of PF among PF talents defined as the top 10 boys and the top 10 girls was analyzed in more detail. We found that the PF of talents increased in both sexes during the study, although the rates of change were a bit higher in girls than boys. In grade 4, the boys achieved the highest change rates for push-ups. The girls achieved the highest change rates in sit-ups and the stand-and-reach test. In grade 7, the largest increases were observed in push-ups (boys) and sit-ups (girls). Overall, the effects were stronger in grade 7 than in grade 4.

### 4.1. PF Talents

The increase in PF among the top 10 boys and girls, especially in grade 7, can be interpreted as a success for the sport schools because they are interested in attracting good athletes to their institutions. The observed increase in PF can be traced to several influential factors. The quality and content of overall physical education at the sport schools may have improved, as well as better identification of PF talent. For example, an increase in experience of the responsible persons at sports school makes facilitates the attraction of high-performance PF talents. Through coaches employed at the school, the quality of physical education and the care of athletes has improved.

The identification of PF talents may have also improved simply because more students applied to sport schools and new schools were integrated (increasing *N* over time). Post hoc analyses of covariance showed that adding the number of tested participants as a covariate reduces the variance in the PF of the top 10, which can be explained by the cohort but does not nullify it. For example, regarding the top 10 PF talents in grade 4, variance explained (p.Eta²) decreased from 0.513 to 0.378 (*p* < 0.01) among boys and from 0.474 to 0.156 (*p* < 0.01) among girls. In grade 7, p.Eta² decreased from 0.686 to 0.457 (*p* < 0.01) among boys and from 0.694 to 0.298 (*p* < 0.01) among girls. The variance explained by the cohort is larger in boys than in girls.

An important point to consider in talent screening is the relative age effect (RAE). RAE refers to chronological age differences between individuals within annually age-grouped cohorts. Children who were born earlier within a cohort have advantages over those who were born later. RAE in sports is a worldwide phenomenon and exists in many, but not all, competitive sports [15]. Data from the current study show an RAE; children born earlier (relative to the test date) are over-represented in the top 10: 32 out of 100 boys (32%) and 39 out of 100 girls (39%) from grade 4 were born in the first quarter. In grade 7, 26 out of 80 boys (32.5%) and 32 out of 80 girls (40%) were born in the first three months of the calendar year. 

Regarding the stability of PF over time, our study shows that 15 out of 70 boys (21.4%) and 12 out of 70 girls (17.1%) reached the top 10 in grades 4 and 7. This indicates that PF shows only moderate stability over time around puberty [16]. The range in variability between adolescents of the same chronological age in physical and anthropometric characteristics reached a maximum around the adolescent growth spurt. These circumstances underline the necessity of steady, periodical talent screening that also considers maturity. A noninvasive method to determine the biological age that is suitable for field testing was provided by Mirwald et al. [17]. They used the known differential timings of growth of height, sitting, and leg length, and expected that the changing relationship between leg length and sitting height with growth may provide an indication of maturational status [17]. The assessment of biological age can further improve talent diagnostics and prevent the sorting of children and adolescents with a lower biological age compared to their classmates. 

### 4.2. Strength and Limitations of the Study

We examined the PF of more than 14,000 children and adolescents (grade 4: 11,451; grade 7: 2614) across 10 years in grade 4 and 8 years in grade 7. Additionally, the test data should be high quality because the FoSS conducted training for test instructors as well as provided supervision on the first day of testing at each school, and the DMT is a quality-proven test battery that allows a standardized assessment of PF [4]. 

From a talent perspective, only a single criterion, juvenile performance, was measured in this study. Aspects such as the speed of performance development, use of performance conditions, and load tolerance [3] were not measured. Given inter-individual differences in growth, training may have influenced the selection of talent in our study and development of the nonlinear performance determinants could not be considered [18].

Another aspect from a talent perspective is the age of peak performance in different sports. The age of best performance for events requiring explosive power and speed occurs at a younger age than for events requiring endurance [19]. Thus, non-specific testing of general motor abilities cannot provide sufficient information for sports with a low maximum performance age and sport-specific training. Specific test instruments need to be used to check relevant characteristics in specific sports [5].

We found a relative age effect in the present data regarding the top talents, which is currently not solved by the sport school system in NRW and its cohort recruitment of pupils. We recommend using the biological age to further define the talent diagnosis criteria in German sport schools.

In the course of the study, sport schools differed in terms of talent promotion, catchment areas, and applicable numbers. In the future, more stable samples will be available because all 18 sports schools in NRW have become steady partners of the project. Lastly, we the results are not generalizable without limitations. Four different types of sport schools exist in Germany consisting of 115 systems of schools linked with competitive sports. These 115 systems differ largely in terms of the basic philosophy and content-based orientation [20].

## 5. Conclusions

The PF of the top 10 boys and girls increased over the course of the study in both grade levels. The improvements were more pronounced in grade 7 than in grade 4, and differences between cohorts were larger in girls than in boys. The overall PF of tested participants remained stable in grade 4 and decreased in grade 7.

The increase in PF of the top 10 boys and girls was only partly explained by an increase in the number of tested individuals over the course of the study. Schools appear to have optimized the concepts of talent identification and promotion. By creating one athletics coach job and one trainer-teacher job for each of the 18 sport schools, PF is likely to further increase.

## Figures and Tables

**Table 1:** Overall *Z*-scores of physical fitness (PF) and regression coefficients of the total sample and the top 10 talents in grades 4 and 7.

Cohort	Grade 4	All	Top 10	Grade 7	All	Top 10
		mean *Z* ± SD	mean *Z* ± SD		mean *Z* ± SD	mean *Z* ± SD
07/08	male (*N* = 226)	108.3 ± 5.1	118.4 ± 1.7			
	female (*N* = 136)	108.8 ± 5.2	117.9 ± 1.6			
	∑ (*N* = 362)	108.5 ± 5.2	118.1 ± 1.6			
08/09	male (*N* = 408)	108.3 ± 5.3	117.8 ± 0.8			
	female (*N* = 276)	108.4 ± 5.5	120.3 ± 1.3			
	∑ (*N* = 684)	108.3 ± 5.3	119.1 ± 1.7			
09/10	male (*N* = 360)	109.5 ± 5.1	120.6 ± 1.7	m (*N* = 30)	108.2 ± 3.8	112.8 ± 2.2
	female (*N* = 207)	109.2 ± 5.5	119.0 ± 1.8	f (*N* = 24)	109.1 ± 4.9	113.9 ± 2.1
	∑ (*N* = 567)	109.4 ± 5.2	119.8 ± 1.9	∑ (*N* = 54)	108.6 ± 4.3	113.3 ± 2.2
10/11	male (*N* = 335)	108.5 ± 5.3	118.9 ± 1.4	m (*N* = 108)	110.1 ± 4.3	117.5 ± 0.6
	female (*N* = 199)	109.9 ± 5.4	120.3 ± 2.0	f (*N* = 75)	111.2 ± 3.7	117.3 ± 1.2
	∑ (*N* = 534)	109.0 ± 5.4	119.6 ± 1.8	∑ (*N* = 183)	110.6 ± 4.1	117.4 ± 0.9
11/12	male (*N* = 529)	107.4 ± 6.9	120.5 ± 1.6	m (*N* = 132)	109.6 ± 5.0	118.0 ± 1.2
	female (*N* = 266)	107.5 ± 7.6	119.7 ± 1.2	f (*N* = 78)	109.3 ± 4.6	115.6 ± 0.9
	∑ (*N* = 795)	107.4 ± 7.2	120.1 ± 1.5	∑ (*N* = 210)	109.5 ± 4.8	116.8 ± 1.7
12/13	male (*N* = 773)	108.0 ± 6.3	121.3 ± 1.3	m (*N* = 131)	108.8 ± 5.1	117.2 ± 1.2
	female (*N* = 425)	106.9 ± 6.9	120.6 ± 1.0	f (*N* = 78)	108.7 ± 5.8	116.9 ± 1.4
	∑ (*N* = 1198)	107.6 ± 6.5	121.0 ± 1.2	∑ (*N* = 209)	108.8 ± 5.4	117.0 ± 1.3
13/14	male (*N* = 997)	108.1 ± 5.5	119.6 ± 0.8	m (*N* = 114)	110.1 ± 4.6	118.1 ± 1.3
	female (*N* = 515)	108.4 ± 6.2	120.6 ± 1.3	f (*N* = 82)	110.8 ± 5.0	117.8 ± 1.5
	∑ (N = 1512)	108.2 ± 5.8	120.1 ± 1.2	∑ (*N* = 196)	110.4 ± 4.8	117.9 ± 1.4
14/15	male (*N* = 1176)	107.7 ± 5.8	120.7 ± 1.2	m (*N* = 273)	105.4 ± 7.7	119.0 ± 2.3
	female (*N* = 609)	107.9 ± 6.9	121.1 ± 0.8	f (*N* = 163)	106.5 ± 8.2	118.6 ± 1.3
	∑ (*N* = 1785)	107.7 ± 6.2	120.9 ± 1.0	∑ (*N* = 436)	105.8 ± 7.9	118.8 ± 1.8
15/16	male (*N* = 1385)	108.6 ± 5.4	120.6 ± 0.8	m (*N* = 310)	107.3 ± 7.5	119.4 ± 1.3
	female (*N* = 705)	108.7 ± 6.5	122.3 ± 0.8	f (*N* = 221)	107.6 ± 8.1	119.9 ± 1.2
	∑ (*N* = 2090)	108.7 ± 5.8	121.5 ± 1.1	∑ (*N* = 531)	107.4 ± 7.7	119.7 ± 1.2
16/17	male (*N* = 1252)	108.4 ± 6.0	121.6 ± 0.5	m (*N* = 444)	108.5 ± 6.2	119.9 ± 0.9
	female (*N* = 619)	108.8 ± 6.6	121.6 ± 0.8	f (*N* = 270)	109.4 ± 6.8	120.2 ± 1.1
	∑ (*N* = 1871)	108.5 ± 6.2	121.6 ± 0.7	∑ (*N* = 714)	108.8 ± 6.5	120.0 ± 1.0
Rate of change (β = Z × year^−1^)	male *N* = 7441)	−0.01	0.32 **	m (*N* = 1542)	−0.29 **	0.76 **
	female (*N* = 3957)	0.00	0.36 **	f (*N* = 991)	−0.24 *	0.80 **
	∑ (*N* = 11,398)	0.00	0.34 **	∑ (*N* = 2553)	−0.27 **	0.78 **
ANCOVA: Effect of cohort (controlled for *N*) Variance explained (p.Eta^2^)	male: *F* =	5.71 (6.42)	10.52 (6.84)	m: F =	12.40 (13.05)	22.44 (10.08)
	*p* =	<0.01 (<0.01)	<0.01 (<0.01)	*p* =	<0.01 (<0.01)	<0.01 (<0.01)
	p.Eta^2^ =	0.007 (0.007)	0.513 (0.378)	p.Eta^2^ =	0.054 (0.049)	0.054 (0.049)
	female: *F* =	5.51 (6.19)	9.02 (2.08)	f: F =	6.29 (6.81)	23.33 (5.10)
	*p* =	<0.01 (<0.01)	<0.01 (0.046)	*p* =	<0.01 (<0.01)	<0.01 (<0.01)
	p.Eta^2^ =	0.012 (0.012)	0.474 (0.156)	p.Eta^2^ =	0.043 (0.040)	0.694 (0.298)

Abbrevation: ** *p* = < 0.01; * *p* = < 0.05.

## Appendix A

**Table A1:** *Z* scores of eight tasks of the top 10 boys and top 10 girls in grade 4.

Cohort	Task	20 m	Long Jump	Balancing	Sit-Ups	Push-ups	Jumping	Stand + Reach	6 min Run
		mean *Z* ± SD	mean *Z* ± SD	mean *Z* ± SD	mean *Z* ± SD	mean *Z* ± SD	mean *Z* ± SD	mean *Z* ± SD	mean *Z* ± SD
07/08	male (*N* = 10)	120.1 ± 5.3	118.4 ± 6.2	116.6 ± 3.1	118.6 ± 5.8	119.1 ± 10.6	122.8 ± 8.4	111.6 ± 5.8	119.7 ± 7.0
	female(*N* = 10)	118.4 ± 4.3	115.9 ± 7.3	114.4 ± 3.8	116.9 ± 5.0	123.0 ± 5.8	125.2 ± 5.7	110.0 ± 10.9	119.5 ± 5.6
	∑ (*N* = 20)	119.3 ± 4.8	117.2 ± 6.7	115.5 ± 3.6	117.8 ± 5.3	121.1 ± 8.6	124.0 ± 7.1	110.8 ± 8.5	119.6 ± 6.2
08/09	male (*N* = 10)	116.6 ± 5.0	113.9 ± 8.4	117.9 ± 6.0	115.5 ± 7.6	121.1 ± 8.9	125.6 ± 5.6	113.4 ± 7.6	118.6 ± 3.6
	female (*N* = 10)	119.7 ± 5.7	120.7 ± 6.8	116.5 ± 2.8	119.3 ± 7.4	122.1 ± 8.0	127.2 ± 6.1	116.8 ± 6.7	119.9 ± 5.9
	∑ (*N* = 20)	118.2 ± 5.5	117.3 ± 8.2	117.2 ± 4.7	117.4 ± 7.6	121.6 ± 8.3	126.4 ± 5.7	115.1 ± 7.2	119.3 ± 4.8
09/10	male (*N* = 10)	119.5 ± 4.8	122.2 ± 6.4	116.7 ± 3.5	117.3 ± 7.2	125.4 ± 5.0	124.9 ± 5.7	116.3 ± 6.1	122.2 ± 5.8
	female (*N* = 10)	118.1 ± 5.3	118.4 ± 6.3	115.0 ± 4.7	116.6 ± 6.6	123.8 ± 7.5	125.4 ± 7.2	115.4 ± 7.5	119.6 ± 5.1
	∑ (*N* = 20)	118.8 ± 5.0	120.3 ± 6.5	115.9 ± 4.1	117.0 ± 6.7	124.6 ± 6.3	125.2 ± 5.9	115.9 ± 6.7	120.9 ± 5.5
10/11	male (*N* = 10)	119.9 ± 5.8	119.4 ± 4.4	114.8 ± 4.8	115.4 ± 4.9	124.4 ± 7.7	125.8 ± 3.8	110.1 ± 4.7	121.1 ± 6.6
	female (*N* = 10)	114.5 ± 4.8	121.3 ± 6.0	115.4 ± 3.0	116.4 ± 5.8	127.7 ± 3.3	127.5 ± 3.0	116.5 ± 6.2	123.3 ± 5.4
	∑ (*N* = 20)	117.2 ± 5.9	120.4 ± 5.2	115.1 ± 3.9	115.9 ± 5.3	126.1 ± 6.0	126.7 ± 3.4	113.3 ± 6.3	122.2 ± 6.0
11/12	male (*N* = 10)	119.2 ± 4.2	115.4 ± 3.8	116.7 ± 5.6	121.1 ± 4.9	127.0 ± 6.9	127.4 ± 2.8	116.8 ± 6.2	120.1 ± 5.2
	female (*N* = 10)	116.2 ± 8.8	119.9 ± 5.0	115.2 ± 4.5	116.1 ± 6.0	125.4 ± 4.8	128.3 ± 3.2	115.2 ± 8.2	121.0 ± 3.6
	∑ (*N* = 20)	117.7 ± 6.9	117.7 ± 4.9	116.0 ± 5.0	118.6 ± 5.9	126.2 ± 5.9	127.9 ± 3.0	116.0 ± 7.1	120.6 ± 4.4
12/13	male (*N* = 10)	120.8 ± 3.8	117.3 ± 3.3	119.0 ± 3.5	122.7 ± 4.4	126.2 ± 4.5	129.1 ± 1.9	113.8 ± 7.5	121.8 ± 5.8
	female (*N* = 10)	120.2 ± 3.2	116.7 ± 4.0	116.8 ± 1.9	121.0 ± 8.8	124.2 ± 4.2	129.0 ± 1.9	114.4 ± 8.8	122.3 ± 4.3
	∑ (*N* = 20)	120.5 ± 3.5	117.0 ± 3.6	117.9 ± 3.0	121.9 ± 6.8	125.2 ± 4.4	129.1 ± 1.9	114.1 ± 8.0	122.1 ± 5.0
13/14	male (*N* = 10)	113.6 ± 4.0	118.2 ± 5.8	116.5 ± 4.2	122.0 ± 7.1	128.9 ± 2.0	126.2 ± 3.8	114.8 ± 9.3	116.9 ± 4.4
	female (*N* = 10)	116.0 ± 5.8	122.8 ± 3.3	114.4 ± 3.1	118.4 ± 7.8	122.1 ± 7.0	126.6 ± 4.8	118.7 ± 6.3	125.9 ± 4.7
	∑ (*N* = 20)	114.8 ± 5.0	120.5 ± 5.2	115.5 ± 3.8	120.2 ± 7.5	125.5 ± 6.1	126.4 ± 4.2	116.8 ± 8.0	121.4 ± 6.4
14/15	male (*N* = 10)	115.3 ± 4.7	118.1 ± 3.1	118.5 ± 3.4	122.5 ± 7.4	128.5 ± 2.9	126.4 ± 5.6	117.0 ± 6.1	119.4 ± 5.7
	female (*N* = 10)	118.0 ± 6.3	119.5 ± 5.9	118.0 ± 6.3	122.7 ± 7.6	122.4 ± 7.6	128.6 ± 3.1	118.7 ± 6.7	121.2 ± 5.5
	∑ (*N* = 20)	116.7 ± 5.6	118.8 ± 4.6	118.3 ± 3.0	122.6 ± 7.3	125.5 ± 6.4	127.5 ± 4.6	117.9 ± 6.3	120.3 ± 5.5
15/16	male (*N* = 10)	115.3 ± 4.4	117.1 ± 7.8	117.2 ± 5.3	122.2 ± 3.9	125.0 ± 5.0	129.0 ± 1.4	114.8 ± 5.4	124.4 ± 6.3
	female (*N* = 10)	118.1 ± 5.5	123.4 ± 5.2	116.6 ± 2.6	121.9 ± 6.0	125.3 ± 4.7	129.5 ± 1.6	119.0 ± 7.8	124.5 ± 2.6
	∑ (*N* = 20)	116.7 ± 5.1	120.3 ± 7.2	116.9 ± 4.1	122.1 ± 5.0	125.2 ± 4.7	129.3 ± 1.5	116.9 ± 6.9	124.5 ± 4.7
16/17	male (*N* = 10)	115.5 ± 3.6	120.1 ± 4.6	120.6 ± 0.8	120.1 ± 7.7	127.0 ± 2.8	128.8 ± 2.5	119.2 ± 4.8	121.7 ± 6.4
	female (*N* = 10)	114.4 ± 5.6	122.0 ± 6.5	116.3 ± 1.7	122.0 ± 5.0	128.6 ± 2.1	127.7 ± 4.4	117.5 ± 8.3	124.4 ± 4.2
	∑ (*N* = 20)	115.0 ± 4.6	121.1 ± 5.5	118.5 ± 2.6	121.1 ± 6.4	127.8 ± 2.6	128.3 ± 3.5	118.4 ± 6.7	123.1 ± 5.4
Rate of change (β = Z × year^−1)^	male (*N* = 100)	−0.54 **	0.09	0.29	0.65 **	0.77 **	0.54 **	0.56 *	0.20
	female (*N* = 100)	−0.24	0.49 *	0.19	0.64 **	0.29	0.32 *	0.64 *	0.57
	∑ (*N* = 200)	−0.39 **	0.29 *	0.24 *	0.65 **	0.53 **	0.43 **	0.60 **	0.39 **

Abbrevation: ** *p* = < 0.01; * *p* = < 0.05.

**Table A2:** Z-scores of eight tasks of the top 10 boys and top 10 girls of grade 7.

Cohort	Task	20 m	Long Jump	Balancing	Sit-Ups	Push-Ups	Jumping	Stand + Reach	6 min Run
		mean *Z* ± SD	mean *Z* ± SD	mean *Z* ± SD	mean *Z* ± SD	mean *Z* ± SD	mean *Z* ± SD	mean *Z* ± SD	mean *Z* ± SD
09/10	male (*N* = 10)	108.4 ± 3.4	113.0 ± 7.5	111.9 ± 5.2	115.2 ± 6.0	114.5 ± 9.3	122.7 ± 7.8	104.8 ± 9.5	111.5 ± 5.0
	female (*N* = 10)	106.5 ± 5.5	113.6 ± 6.4	112.8 ± 3.1	112.9 ± 4.8	110.7 ± 10.6	122.5 ± 7.1	115.4 ± 10.1	116.5 ± 4.2
	∑ (*N* = 20)	107.5 ± 4.6	113.3 ± 6.8	112.4 ± 4.2	114.1 ± 5.4	112.6 ± 9.9	122.6 ± 7.2	110.1 ± 11.0	114.0 ± 5.2
10/11	male (*N* = 10)	122.1 ± 5.4	121.7 ± 5.4	116.0 ± 2.4	119.6 ± 7.8	113.1 ± 5.1	124.9 ± 5.2	109.4 ± 7.3	113.0 ± 3.9
	female (*N* = 10)	115.7 ± 6.1	119.8 ± 5.3	114.0 ± 1.9	113.7 ± 5.4	115.2 ± 3.2	127.5 ± 3.2	111.4 ± 10.8	121.3 ± 4.4
	∑ (*N* = 20)	118.9 ± 6.5	120.8 ± 5.3	115.0 ± 2.4	116.7 ± 7.2	114.2 ± 4.3	126.2 ± 4.4	110.4 ± 9.0	117.2 ± 5.9
11/12	male (*N* = 10)	118.4 ± 3.6	118.9 ± 7.0	115.1 ± 3.1	116.6 ± 7.9	122.8 ± 5.9	128.1 ± 2.6	109.5 ± 7.2	114.9 ± 4.3
	female (*N* = 10)	114.0 ± 7.0	119.3 ± 6.0	112.9 ± 4.7	114.0 ± 5.4	109.5 ± 5.3	128.3 ± 2.9	111.5 ± 8.9	115.0 ± 7.2
	∑ (*N* = 20)	116.2 ± 5.9	119.1 ± 6.4	114.0 ± 4.0	115.3 ± 6.7	116.2 ± 8.7	128.2 ± 2.6	110.5 ± 8.0	115.0 ± 5.8
12/13	male (*N* = 10)	112.8 ± 4.5	122.6 ± 6.4	114.6 ± 3.8	117.6 ± 7.3	119.5 ± 6.1	125.5 ± 4.5	114.0 ± 7.2	110.6 ± 5.1
	female (*N* = 10)	113.7 ± 2.7	120.6 ± 4.7	113.0 ± 4.3	118.6 ± 2.4	114.1 ± 11.7	125.0 ± 3.6	115.2 ± 7.8	115.0 ± 5.2
	∑ (*N* = 20)	113.3 ± 3.6	121.6 ± 5.6	113.8 ± 4.0	118.1 ± 5.3	116.8 ± 9.5	125.3 ± 3.9	114.6 ± 7.3	112.8 ± 5.5
13/14	male (*N* = 10)	119.0 ± 8.7	119.8 ± 5.3	116.4 ± 1.7	118.5 ± 5.1	120.7 ± 5.3	129.0 ± 1.6	107.2 ± 8.3	113.9 ± 5.6
	female (*N* = 10)	117.4 ± 6.3	121.7 ± 3.9	113.5 ± 2.3	119.5 ± 6.7	113.9 ± 7.4	128.6 ± 2.1	109.7 ± 10.5	118.3 ± 4.9
	∑ (*N* = 20)	118.2 ± 7.4	120.8 ± 4.6	115.0 ± 2.5	119.0 ± 5.8	117.3 ± 7.2	128.8 ± 1.8	108.5 ± 9.3	116.1 ± 5.6
14/15	male (*N* = 10)	121.7 ± 5.8	119.4 ± 7.7	116.6 ± 2.2	121.8 ± 6.2	118.5 ± 9.3	128.7 ± 3.1	111.4 ± 8.1	113.8 ± 3.2
	female (*N* = 10)	112.5 ± 6.5	122.8 ± 6.0	112.0 ± 5.6	120.9 ± 4.3	114.0 ± 8.4	128.7 ± 2.8	120.6 ± 6.1	116.9 ± 6.0
	∑ (*N* = 20)	117.1 ± 7.6	121.1 ± 6.9	114.3 ± 4.8	121.4 ± 5.2	116.3 ± 8.9	128.7 ± 2.9	116.0 ± 8.4	115.4 ± 4.9
15/16	male (*N* = 10)	119.3 ± 5.6	120.2 ± 6.4	114.2 ± 3.9	122.4 ± 6.6	123.9 ± 7.7	125.8 ± 5.4	112.1 ± 3.7	117.6 ± 4.7
	female (*N* = 10)	112.0 ± 4.4	118.5 ± 2.3	114.8 ± 0.4	125.2 ± 5.4	121.6 ± 9.3	129.2 ± 2.2	122.2 ± 7.0	115.5 ± 5.1
	∑ (*N* = 20)	115.7 ± 6.2	119.4 ± 4.8	114.5 ± 2.7	123.8 ± 6.0	122.8 ± 8.4	127.5 ± 4.4	117.2 ± 7.5	116.6 ± 4.9
16/17	male (*N* = 10)	114.2 ± 3.7	118.0 ± 7.8	116.2 ± 2.3	124.5 ± 5.7	127.7 ± 2.7	127.9 ± 4.0	116.6 ± 6.3	114.3 ± 7.4
	female (*N* = 10)	109.8 ± 3.7	119.9 ± 5.3	113.8 ± 2.4	124.7 ± 5.4	126.7 ± 4.9	126.8 ± 4.2	118.1 ± 5.4	121.5 ± 2.7
	∑ (*N* = 20)	112.0 ± 4.2	119.0 ± 6.6	115.0 ± 2.6	124.6 ± 5.4	127.1 ± 3.8	127.4 ± 4.0	117.4 ± 5.8	117.9 ± 6.5
Rate of change (β = *Z* × year^−1^)	male (*N* = 80)	0.51	0.32	0.33	+1.14 **	+1.58 **	0.55 *	+1.13 **	0.51 *
	female (*N* = 80)	0.05	0.59	0.11	+1.93 **	+1.87 **	0.52 *	+1.13 *	0.18
	∑ (*N* = 160)	0.28	0.45 *	0.22	+1.53 **	+1.73 **	0.53 **	+1.13 **	0.34

Abbrevation: ** *p* = < 0.01; * *p* = < 0.05.

**Table A3:** Z-scores of the eight different tasks of all test participants in grade 4.

Cohort	Task	20 m	Long Jump	Balancing	Sit-Ups	Push-Ups	Jumping	Stand + Reach	6 min Run
		mean *Z* ± SD	mean *Z* ± SD	mean *Z* ± SD	mean *Z* ± SD	mean *Z* ± SD	mean *Z* ± SD	mean *Z* ± SD	mean *Z* ± SD
07/08	male (*N* = 226)	111.3 ± 6.7	106.2 ± 7.6	107.8 ± 8.9	104.9 ± 9.0	105.4 ± 9.5	115.3 ± 8.8	105.1 ± 8.1	110.6 ± 7.6
	female (*N* = 136)	109.7 ± 7.7	108.2 ± 7.9	108.1 ± 7.4	104.4 ± 9.9	106.9 ± 9.6	116.2 ± 9.5	104.4 ± 7.9	112.1 ± 7.8
	∑ (*N* = 362)	110.7 ± 7.1	106.9 ± 7.7	107.9 ± 8.4	104.7 ± 9.3	105.9 ± 9.5	115.6 ± 9.0	104.9 ± 8.0	111.2 ± 7.7
08/09	male (*N* = 408)	109.6 ± 7.2	105.1 ± 7.8	108.1 ± 8.8	106.5 ± 8.5	109.6 ± 11.1	113.3 ± 9.9	104.1 ± 9.0	109.6 ± 7.3
	female (*N* = 276)	108.4 ± 7.7	107.6 ± 8.1	108.5 ± 7.9	105.3 ± 8.9	107.9 ± 11.4	114.7 ± 10.2	104.2 ± 8.8	110.7 ± 7.9
	∑ (*N* = 684)	109.1 ± 7.5	106.1 ± 8.0	108.3 ± 8.5	106.0 ± 8.7	108.9 ± 11.2	113.9 ± 10.0	104.1 ± 8.9	110.1 ± 7.6
09/10	male (*N* = 360)	110.6 ± 7.3	107.3 ± 8.2	108.3 ± 8.6	105.7 ± 7.6	111.0 ± 10.0	116.2 ± 9.5	105.1 ± 8.5	112.3 ± 7.3
	female (*N* = 207)	108.8 ± 7.9	107.2 ± 8.9	108.7 ± 7.5	104.7 ± 8.4	108.5 ± 12.0	118.8 ± 9.7	104.7 ± 9.5	112.3 ± 8.7
	∑ (*N* = 567)	109.9 ± 7.5	107.3 ± 8.5	108.4 ± 8.2	105.4 ± 7.9	110.1 ± 10.8	117.1 ± 9.7	105.0 ± 8.9	112.3 ± 7.9
10/11	male (*N* = 335)	109.4 ± 7.4	105.7 ± 7.9	108.9 ± 8.6	105.0 ± 7.4	109.7 ± 10.4	113.4 ± 10.4	104.7 ± 8.8	111.1 ± 8.0
	female (*N* = 199)	108.1 ± 7.0	107.9 ± 8.6	109.2 ± 7.6	105.3 ± 7.6	111.7 ± 11.4	117.0 ± 10.5	106.4 ± 8.4	113.6 ± 7.9
	∑ (*N* = 534)	108.9 ± 7.3	106.6 ± 8.2	109.0 ± 8.2	105.1 ± 7.5	110.5 ± 10.8	114.7 ± 10.6	105.3 ± 8.7	112.0 ± 8.0
11/12	male (*N* = 529)	109.4 ± 9.1	104.3 ± 9.3	108.6 ± 9.1	106.7 ± 9.2	107.5 ± 12.8	111.0 ± 11.2	103.1 ± 9.4	108.5 ± 9.6
	female (*N* = 266)	106.2 ± 9.8	105.4 ± 9.8	108.2 ± 8.2	107.2 ± 10.2	107.6 ± 14.9	113.1 ± 12.2	103.3 ± 9.9	109.4 ± 10.4
	∑ (*N* = 795)	108.3 ± 9.5	104.7 ± 9.5	108.5 ± 8.8	106.9 ± 9.5	107.5 ± 13.5	111.7 ± 11.5	103.2 ± 9.6	108.8 ± 9.9
12/13	male (*N* = 773)	109.0 ± 8.6	104.4 ± 8.8	107.5 ± 8.9	106.3 ± 9.6	111.5 ± 10.8	112.8 ± 10.3	103.2 ± 9.3	109.5 ± 8.6
	female (*N* = 425)	105.7 ± 9.1	104.2 ± 9.9	107.3 ± 8.5	103.9 ± 10.0	109.3 ± 11.3	112.5 ± 12.0	103.0 ± 10.0	109.6 ± 9.4
	∑ (*N* = 1198)	107.8 ± 8.9	104.3 ± 9.2	107.4 ± 8.8	105.5 ± 9.8	110.7 ± 11.0	112.7 ± 10.9	103.2 ± 9.5	109.5 ± 8.9
13/14	male (*N* = 997)	106.5 ± 7.8	104.9 ± 8.5	109.3 ± 8.7	105.7 ± 8.6	108.6 ± 13.0	115.1 ± 10.1	103.8 ± 9.3	110.6 ± 8.1
	female (*N* = 515)	105.2 ± 8.7	106.9 ± 9.5	109.5 ± 8.3	105.9 ± 9.1	107.3 ± 12.3	116.4 ± 10.8	104.1 ± 10.0	112.0 ± 9.3
	∑ (*N* = 1512)	106.1 ± 8.2	105.6 ± 8.9	109.3 ± 8.6	105.7 ± 8.8	108.1 ± 12.8	115.5 ± 10.3	103.9 ± 9.5	111.1 ± 8.5
14/15	male (*N* = 1176)	106.9 ± 7.6	104.4 ± 8.7	108.6 ± 8.9	105.5 ± 9.2	107.2 ± 12.6	113.9 ± 10.8	104.6 ± 8.7	110.1 ± 8.2
	female (*N* = 609)	105.3 ± 9.0	105.1 ± 10.2	108.7 ± 8.9	105.4 ± 9.7	108.2 ± 12.6	115.2 ± 11.3	104.5 ± 10.2	110.5 ± 9.6
	∑ (*N* = 1785)	106.4 ± 8.2	104.7 ± 9.2	108.6 ± 8.9	105.4 ± 9.3	107.5 ± 12.6	114.3 ± 11.0	104.6 ± 9.2	110.2 ± 8.7
15/16	male (*N* = 1385)	107.7 ± 8.7	105.3 ± 8.1	108.3 ± 9.0	106.3 ± 8.4	109.0 ± 11.8	116.4 ± 9.6	105.0 ± 9.1	110.9 ± 8.2
	female (*N* = 705)	106.4 ± 7.9	106.7 ± 9.6	108.4 ± 8.5	105.5 ± 9.7	107.6 ± 12.8	116.7 ± 10.3	105.9 ± 10.2	112.5 ± 9.4
	∑ (*N* = 2090)	107.2 ± 7.2	105.8 ± 8.6	108.3 ± 8.8	106.0 ± 8.9	108.5 ± 12.1	116.5 ± 9.9	105.3 ± 9.5	111.5 ± 8.6
16/17	male (*N* = 1252)	106.8 ± 7.1	104.8 ± 8.6	108.7 ± 9.0	106.8 ± 9.0	106.9 ± 13.0	116.6 ± 10.7	105.2 ± 9.0	111.4 ± 8.6
	female (*N* = 619)	105.6 ± 7.7	106.9 ± 9.7	109.2 ± 7.8	106.4 ± 10.0	106.8 ± 13.6	117.2 ± 10.9	105.9 ± 9.4	112.2 ± 9.8
	∑ (*N* = 1871)	106.4 ± 7.3	105.5 ± 9.0	108.8 ± 8.6	106.7 ± 9.4	106.8 ± 13.2	116.8 ± 10.8	105.4 ± 9.1	111.7 ± 9.0
Rate of change (β = *Z* × year^−1^)	male *N* = 7441)	−0.46 **	−0.11 **	0.05	0.08 *	−0.22 **	0.37 **	0.11 **	0.12 **
	female (*N* = 3957)	−0.38 **	−0.09	0.06	0.12 *	−0.17 *	0.16 *	0.19 **	0.10
	∑ (*N* = 11,398)	−0.42 **	−0.11 **	0.06	0.10 **	−0.20 **	0.29 **	0.14 **	0.10 **

Abbrevation: ** *p* = < 0.01; * *p* = < 0.05.

**Table A4:** *Z* scores of the eight different tasks of all test participants in grade 7.

Cohort	Task	20 m	Long Jump	Balancing	Sit-Ups	Push-Ups	Jumping	Stand + Reach	6 min Run
		mean *Z* ± SD	mean *Z* ± SD	mean *Z* ± SD	mean *Z* ± SD	mean *Z* ± SD	mean *Z* ± SD	mean *Z* ± SD	mean *Z* ± SD
09/10	male (*N* = 30)	103.5 ± 7.6	107.2 ± 9.2	108.6 ± 7.2	111.2 ± 7.7	108.5 ± 9.0	119.3 ± 6.6	100.2 ± 10.3	107.3 ± 5.4
	female (*N* = 24)	101.9 ± 7.7	110.2 ± 6.9	107.4 ± 8.4	110.0 ± 6.7	105.8 ± 12.3	118.0 ± 8.8	108.9 ± 11.5	110.9 ± 6.6
	∑ (*N* = 54)	102.8 ± 7.6	108.5 ± 8.3	108.1 ± 7.7	110.6 ± 7.2	107.3 ± 10.6	118.7 ± 7.6	104.1 ± 11.6	108.9 ± 6.2
10/11	male (*N* = 108)	113.2 ± 7.3	110.6 ± 9.3	110.6 ± 6.7	106.6 ± 8.4	107.4 ± 8.3	119.0 ± 7.3	105.9 ± 8.7	108.0 ± 6.3
	female (*N* = 75)	109.7 ± 6.3	112.2 ± 6.7	109.4 ± 6.3	109.1 ± 6.4	106.7 ± 7.8	120.8 ± 6.7	108.0 ± 7.5	114.0 ± 6.5
	∑ (*N* = 183)	111.7 ± 7.1	111.2 ± 8.4	110.1 ± 6.6	107.6 ± 7.7	107.1 ± 8.0	119.7 ± 7.1	106.7 ± 8.2	110.4 ± 7.0
11/12	male (*N* = 132)	112.1 ± 6.6	109.5 ± 8.3	110.9 ± 6.5	109.2 ± 9.0	107.3 ± 12.0	119.8 ± 8.1	101.7 ± 9.8	106.1 ± 6.9
	female (*N* = 78)	103.5 ± 7.5	112.3 ± 7.7	109.1 ± 6.7	107.4 ± 7.6	100.3 ± 10.8	121.8 ± 7.6	104.7 ± 9.9	111.0 ± 6.4
	∑ (*N* = 210)	110.5 ± 7.0	110.6 ± 8.2	110.2 ± 6.6	108.5 ± 8.5	104.6 ± 12.0	120.6 ± 8.0	102.8 ± 9.9	107.9 ± 7.1
12/13	male (*N* = 131)	108.6 ± 6.7	108.3 ± 9.7	110.8 ± 6.8	109.1 ± 7.2	102.8 ± 14.1	120.2 ± 7.1	103.7 ± 8.6	106.2 ± 7.8
	female (*N* = 78)	103.5 ± 7.5	110.8 ± 8.7	108.7 ± 6.5	110.0 ± 8.1	96.0 ± 15.6	121.1 ± 6.8	108.1 ± 9.4	110.4 ± 6.2
	∑ (*N* = 209)	106.7 ± 7.4	109.3 ± 9.4	110.0 ± 6.8	109.4 ± 7.5	100.2 ± 15.0	120.6 ± 7.0	105.4 ± 9.1	107.8 ± 7.5
13/14	male (*N* = 114)	114.8 ± 7.0	108.9 ± 8.3	109.8 ± 8.0	109.9 ± 9.9	105.3 ± 11.0	120.8 ± 7.4	103.4 ± 8.0	107.3 ± 6.0
	female (*N* = 82)	109.7 ± 7.8	112.1 ± 7.4	109.2 ± 7.0	111.7 ± 8.9	101.7 ± 11.2	122.3 ± 7.6	108.4 ± 10.2	111.3 ± 6.9
	∑ (*N* = 196)	112.7 ± 7.8	110.3 ± 8.1	109.6 ± 7.6	110.7 ± 9.5	103.8 ± 11.2	121.4 ± 7.5	105.5 ± 9.3	109.0 ± 6.7
14/15	male (*N* = 273)	109.5 ± 8.8	104.5 ± 12.2	108.3 ± 9.2	103.1 ± 10.8	97.7 ± 13.0	116.7 ± 11.5	100.0 ± 10.0	103.7 ± 10.2
	female *(N* = 163)	102.9 ± 9.3	106.6 ± 11.8	108.8 ± 7.0	106.1 ± 11.8	95.1 ± 13.5	117.3 ± 12.2	107.1 ± 10.7	107.9 ± 10.9
	∑ (*N* = 436)	107.1 ± 9.5	105.3 ± 12.1	108.5 ± 8.4	104.2 ± 11.3	96.7 ± 13.2	116.9 ± 11.8	102.6 ± 10.8	105.3 ± 10.7
15/16	male (*N* = 310)	107.1 ± 8.7	104.7 ± 11.3	109.1 ± 8.4	105.9 ± 11.2	106.2 ± 15.8	118.0 ± 11.0	102.9 ± 9.6	104.7 ± 10.2
	female (*N* = 221)	102.0 ± 10.9	106.9 ± 11.1	108.7 ± 6.9	106.6 ± 11.2	102.4 ± 14.3	118.5 ± 12.0	107.6 ± 11.0	107.8 ± 11.1
	∑ (*N* = 531)	105.0 ± 10.0	105.6 ± 11.3	108.9 ± 7.8	106.2 ± 11.2	104.6 ± 15.3	118.2 ± 11.4	104.9 ± 10.4	106.0 ± 10.7
16/17	male (*N* = 444)	106.0 ± 7.7	105.3 ± 10.8	109.4 ± 8.1	107.4 ± 9.8	112.2 ± 11.5	119.3 ± 9.4	102.5 ± 9.9	105.5 ± 9.6
	female (*N* = 270)	103.1 ± 8.2	108.4 ± 10.7	107.1 ± 8.7	109.0 ± 9.8	110.5 ± 11.4	119.1 ± 9.7	108.4 ± 10.1	108.8 ± 11.4
	∑ (*N* = 714)	104.9 ± 8.0	106.5 ± 10.9	108.5 ± 8.4	108.0 ± 9.8	111.6 ± 11.5	119.3 ± 9.5	104.8 ± 10.4	106.8 ± 10.5
Rate of change (β = *Z* × year^−1^)	male (*N* = 1542)	−0.95 **	−0.86 **	−0.24 *	−0.35 **	0.80 **	−0.15	−0.17	−0.35 **
	female (N = 991)	−0.86 **	−0.76 **	−0.27 *	−0.17	+1.10 **	−0.39 *	0.17	−0.69 **
	∑ (*N* = 2553)	−0.91 **	−0.82 **	−0.25 **	−0.28 **	0.91 **	−0.25 *	−0.04	−0.49 **

Abbrevation: ** *p* = < 0.01; * *p* = < 0.05.
